# TonB-Dependent Transporters in Sphingomonads: Unraveling Their Distribution and Function in Environmental Adaptation

**DOI:** 10.3390/microorganisms8030359

**Published:** 2020-03-03

**Authors:** Devyani Samantarrai, Annapoorni Lakshman Sagar, Ramurthy Gudla, Dayananda Siddavattam

**Affiliations:** Department of Animal Biology, School of Life Sciences, University of Hyderabad, Hyderabad 500 046, India; devyani66@gmail.com (D.S.); annapoornilakshmansagar@gmail.com (A.L.S.); grmurthygp120@gmail.com (R.G.)

**Keywords:** TonB-dependent transporter (TBDT), sphingomonads, xenobiotics

## Abstract

TonB-dependent transport system plays a critical role in the transport of nutrients across the energy-deprived outer membrane of Gram-negative bacteria. It contains a specialized outer membrane TonB-dependent transporter (TBDT) and energy generating (ExbB/ExbD) and transducing (TonB) inner membrane multi-protein complex, called TonB complex. Very few TonB complex protein-coding sequences exist in the genomes of Gram-negative bacteria. Interestingly, the TBDT coding alleles are phenomenally high, especially in the genomes of bacteria surviving in complex and stressful environments. Sphingomonads are known to survive in highly polluted environments using rare, recalcitrant, and toxic substances as their sole source of carbon. Naturally, they also contain a huge number of TBDTs in the outer membrane. Out of them, only a few align with the well-characterized TBDTs. The functions of the remaining TBDTs are not known. Predictions made based on genome context and expression pattern suggest their involvement in the transport of xenobiotic compounds across the outer membrane.

## 1. Introduction

**Figure 1:**
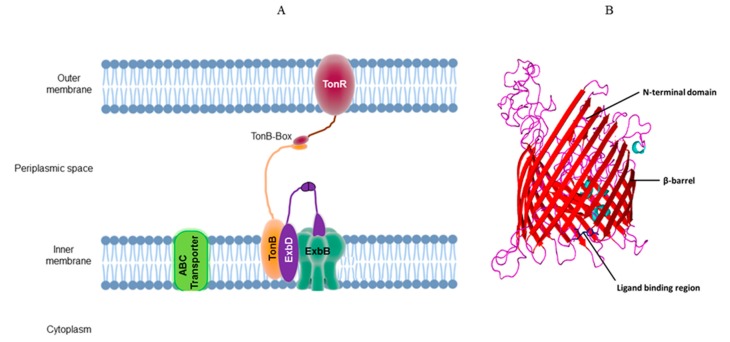
(**A**) Schematic diagram of TonB-dependent transport system. (**B**) Typical structural features of an outer membrane transporter (TBDT), 22 β-barrel structure, N-terminal plug domain, and substrate binding motif are indicated with arrows.

The outer membrane of Gram-negative bacteria performs several important functions. It acts as a barrier to prevent the entry of antibiotics and other toxic chemicals and protects the cell wall by denying access to cell wall degrading enzymes. However, existence of an energy-deprived outer membrane is a hurdle for the uptake of nutrients in Gram-negative bacteria [1]. A majority of nutrients gains entry into periplasmic space by diffusing through the outer membrane via a pore-like structure formed in outer membrane-associated β-barrel containing proteins, otherwise known as porins [2]. However, certain scarcely available nutrients depend on active transport to cross the energy-deprived outer membrane. The active transport mechanism of the outer membrane is known as TonB-dependent transport system. The system contains two components, the inner membrane-associated TonB complex and an outer membrane-associated TonB-dependent transporter (TBDT). The TonB complex contains proton motif force (PMF) components, ExbB/ExbD, and energy transducer TonB in a ratio of 7:2:1 [3]. The TonB complex has unique role in outer membrane transport. The PMF components ExbB/ExbD generate energy by pumping protons across the inner membrane, while TonB transduces this energy to the outer membrane-localized TBDT. The TonB protein contains three domains: the N-terminal transmembrane helix, C-terminal domain, and a proline-rich rigid central domain. The N-terminal region is embedded in the inner membrane and is associated with one of the transmembrane domains of ExbB [4]. The longer C-terminal region extends into periplasmic space and specifically interacts with TonB-box of the outer membrane-localized TBDT. These interactions of TonB and TBDT facilitate the transduction of energy required to transport scarcely available nutrients across the outer membrane. The TBDT possesses unique structural features and exhibits a two-domain structure. The C-terminal domain embedded in the outer membrane contains one of the largest 22-stranded β-barrel with extracellular loops. The N-terminal globular domain, through its unique structural features, establishes cross-talk with the inner membrane-localized TonB complex. An energy coupling consensus pentapeptide motif (ETVIV) designated as “TonB-box” physically interacts with the C-terminal domain of inner membrane-localized, periplasmically exposed TonB [5]. The TBDT undergoes conformational changes upon substrate binding. This conformational change induces structural transition from a state of order to disorder in the TonB-box motif [6]. This disordered state of TonB-box of TBDT is recognized by TonB. The TBDT and TonB interactions are transient, the disordered state of TonB-box returns to an ordered state after completion of substrate transport [7]. TonB plays a critical role in supplying energy required for the structural transition of TBDT. TonB harvests energy generated by PMF components ExbB/ExbD and transduces it to TBDT (Figure 1).

The TonB complex of TBDT is highly conserved among Gram-negative bacteria. The genome sequences of Gram-negative bacteria contain a limited number of alleles to code for TonB complex proteins, TonB and ExbB/ExbD. Though the overall structural features of TBDT are conserved, there exist substantial differences in the residues of ligand binding sites. Such diversity in the residues of ligand binding sites suggests the existence of specialized TBDTs for transport of a variety of scarcely available nutrients in the environment. The copy number of TBDTs shows a very unique pattern among Gram-negative bacteria. Genomes isolated from the cells grown in less stressful environments show existence of a smaller number of TBDT coding alleles. Their numbers in such strains does not exceed four to five alleles per genome. However, the number of alleles coding TBDTs ie more in the genomes of cells isolated from harsh environments [8]. The gut microbiome sequences show an unusually high number of TBDT coding sequences [9]. Similarly, in the genomes of sphingomonads, namely *Sphingobium japonicum*, *Sphingobium indicum*, *Sphingobium fuliginis*, which live in harsh climates, a very high number of TBDT coding alleles are identified. In fact, the number of TBDTs appears to proportionately increase with the complexity of the environment [9]. However, there exists no study to link the increased number of TBDTs and complexity of the habitat. Since sphingomonads survive in habitats polluted with a variety of xenobiotics, in this study we attempt to examine if these TBDTs have a role in the transport of xenobiotic compounds by examining the genome information of certain Sphingomonadaceae members.

## 2. The TonB-Dependent Transport System

TonB-dependent transport system derived its name due to the fact that phage T1 failed to infect the null mutants of *tonA* and *tonB* in *Esterichia coli* [10]. Subsequent studies performed to understand this unusual observation have identified the physiological role of these two genes. The roles of these two genes are now well established. The *tonA* codes for TBDT designated as FhuA and is involved in the transport of ferrichrome [10]. Initial studies gave an impression that TonB-dependent transport system, consisting of FhuA, serves exclusively for iron-siderophore complex uptake. However, recent studies have dismantled this myth and showed the involvement of FhuA in the transport of antibiotics. In addition to ferrichrome, FhuA successfully transported siderophore structural mimic Albomycin and Rifamycin CGP 4832, which have no structural similarity with siderophores [11]. Similarly, the outer membrane transporter, BtuB, of *E. coli* was found to be a member of the TBDT family and interacts with the inner membrane-associated TonB complex to facilitate the active transport of vitamin B_12_ across the outer membrane [12]. Likewise, a wide variety of TBDTs was identified in many pathogenic and non-pathogenic bacteria to translocate a variety of substrates across the outer membrane via TonB-dependent transport system [13]. Transport of nickel complexes in human pathogen *Helicobacter pylori*, maltodextrins in the environmental bacteria *Caulobacter crescentus*, and sucrose in plant pathogen *Xanthomonas campestris, pv. campestris* are certain classical examples that show involvement of the TonB-dependent transport system in the transport of substrates other than iron. Table 1 gives an exhaustive list of substrates predicted to be transported through TonB-dependent transport system.

## 3. TonB-Dependent Transporters (TBDTs) and Environmental Adaptation

Sphingomonads survive in various stressful environments. They survive in a nutrient-poor phyllosphere [14], extremely cold marine waters [15] and highly toxic and polluted environments with metals [16], phenanthrene [17,18,19,20], polyethylene glycol [21], alkylphenols [22], dioxins [23], naphthalene [20], diphenyl ethers [24], organophosphates [25,26] and organochlorides [27,28,29]. Their survival under these stressful conditions depends on their ability to use these unusual substrates, hitherto unknown to natural habitats, as a carbon source (Table 2). Such a task can be accomplished with an efficient catabolic repertoire, an effective transport system. The genome sequences of these strains indeed reveal the existence of a novel catabolic repertoire [26]. Interestingly their genomes also contain an unusually high number of TBDTs.

## 4. Unique TonB Complex in *Sphingobium fuliginis*

Organophosphate (OP)-degrading sphingomonads contain phosphotriesterases (PTE), also known as organophosphate hydrolase (OPH), capable of degrading the third ester linkage found in OP insecticides and nerve agents [30]. The membrane-associated PTE target the membrane in a pre-folded conformation following twin-arginine transport (Tat) pathway. The Tat pathway inserts PTE into the inner membrane-facing periplasmic space of the cell. Recent studies have shown PTE as part of a multiprotein membrane-associated TonB complex. Interestingly, the TonB complex components were co-purified along with PTE. PTE are shown to interact physically with TonB complex components ExbB/ExbD and TonB, showing the existence of a unique four-component TonB complex in *S. fuliginis* [31]. Co-purification of TonB complex components along with PTE and the inability of *pte* null mutants of *Sphingopyxis wildii* to grow in a medium with OP insecticide methyl parathion as a source of phosphate suggest the involvement of a novel TonB-dependent transport system in transport of OP insecticides.

The sphingomonads survive using a variety of organic compounds as a source of carbon and energy [14]. They also contain a rather unusually high number of putative TBDTs when compared to other Gram-negative bacteria surviving in relatively stress-free habitats [32]. Some of these TBDTs are induced when they are grown in the presence of these xenobiotic compounds. In *Sphingomonas alaskensis*, a threefold increase is noticed in the expression of TBDTs to facilitate transport of nutrients in increased viscous water [15]. Sphingomonads like *Sphongobium* sp. BA1, *Sphingobium cupriresistence*, and *Novosphingobium PPIY* can also withstand the stress imposed by metal ions like Ni^2+^, Cu^2+^, and Pb^2+^, respectively, due to increased expression of TBDTs [16]. Differential expression of TBDTs was evident in *Sphingomonas wittichii* RW1 strains grown in the presence of dibenzofuran (DF) and dibenzo-p-dioxin (DD). The substrate-specific induction pattern of TBDTs suggests the existence of substrate-specific TonB-dependent transport systems in sphingomonads [33,34]. Supporting this proposition, the induction of TBDTs involved in transport of alginate was only observed when *Sphingomonas* sps. A1 cells were grown on alginate. These TBDTs directly incorporated alginate molecules into the cytoplasm without degradation [35]. There are 148 TBDTs in *Novosphingobium resinovorans* SA1 (Table 2). One of them showed a seven-fold increased expression when the cells were grown in sulfanilic acid. Such an increase in TBDT amounts is believed to facilitate active transport of polar sulfanilic acid across the outer membrane [36]. TonB-dependent transport system appears to be advantageous to bacteria in more than one way. Since they transport large molecules across the membrane, it helps bacteria to utilize complex substrates as a source of carbon. Further, the existence of substrate-specific transporters facilitates adaptation of sphingomonads by scavenging nutrients that occur at a very low concentration.

## 5. TBDTs of *S. fuliginis*

**Figure 2:**
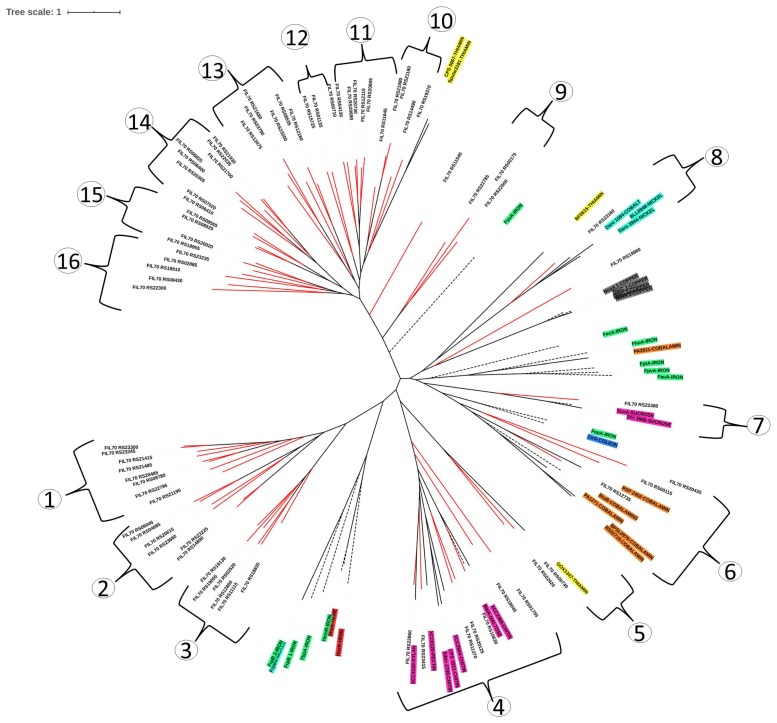
Phylogenetic tree constructed for *_Sf_*TBDTs. The *_Sf_*TBDTs clustered with functionally characterized TBDT sequences are present in clusters 4, 5, 6, 7, and 8. Dashed lines of clades indicate experimentally characterized TBDTs. Red lines of the clade indicate uncharacterized TBDTs from *Sphingobium fuliginis*. TBDTs involved in transport of iron are highlighted with dark green; thiamin in yellow; nickel and cobalt in turquoise; cobalamin in orange; copper in grey; colicin in blue; heme in red; and all carbohydrates in dark pink.

**Figure 3:**
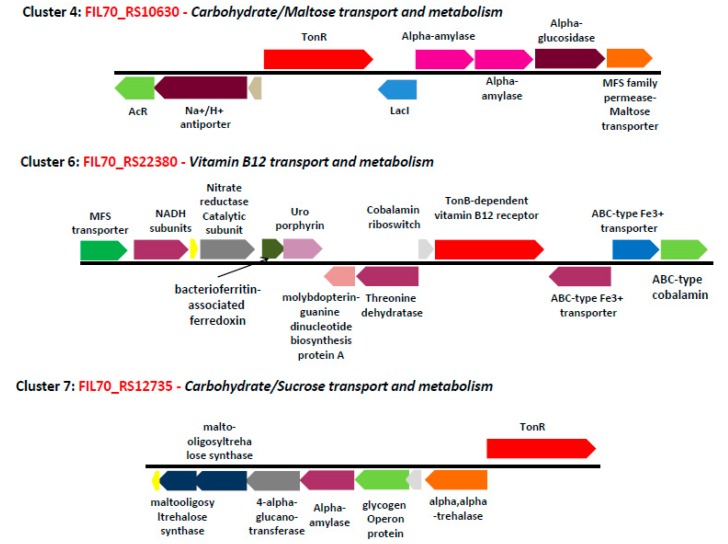
Genome-context analysis of *Sphingobium fuliginis* TBDTs found in clusters 4, 6, and 7.

The fully annotated genome sequence of *S. fuliginis* has shown the existence of more than 100 putative TBDT (*_Sf_*TBDT) coding sequences. Out of these 100, only 75 *_Sf_*TBDTs have shown the existence of a 22-stranded β-barrel and N-terminal plug domain, the typical characteristic feature of a TBDT. A phylogenetic tree was constructed by including these uncharacterized *_Sf_*TBDTs along with TBDTs whose function is either experimentally characterized or predicted based on genome context (Figure 2, Table 1). The phylogenetic tree thus constructed gave 16 TBDT clusters. Of these, only five clusters (cluster numbers 4, 5, 6, 7, and 8) contained TBDTs with known functions. Interestingly, out of 75 putative *_Sf_*TBDTs only 14 clustered with these five known groups of TBDT sequences. The genes coding transporters often coexist with the genes associated with the metabolism of their cognate substrates. They also contain identical promoters and other regulatory elements to ensure common expression and repression pattern in response to a physiological condition. Therefore, such genomic context of a transporter is taken as an indicator of its function [37]. As expected, the genome context of these 14 TBDTs that clustered with TBDTs of known function supports the results obtained through phylogenetic tree (Figure 3).

**Figure 4:**
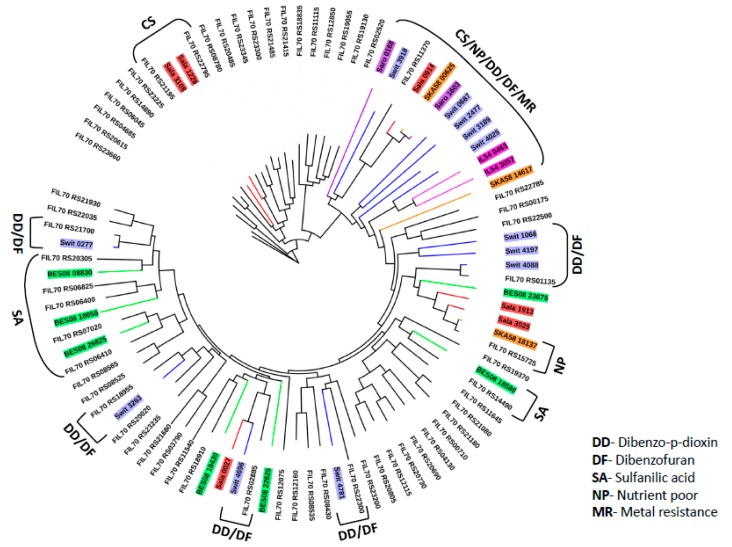
Expression pattern-based phylogenetic tree. Xenobiotic responsive TBDT sequences of *Sphingomonas wittichii* RW1 (blue), *Sphingobium* sp. ba1 (dark pink), *Sphingopyxis alaskensis* RB2256 (red), *Sphingomonas* sp. SKA58 (orange), and *Novosphingobium aromaticivorans* DSM 12444 (purple) are included along with 61 uncharacterized *_Sf_*TBDTs (black) while constructing the phylogenetic tree.

**Figure 5:**
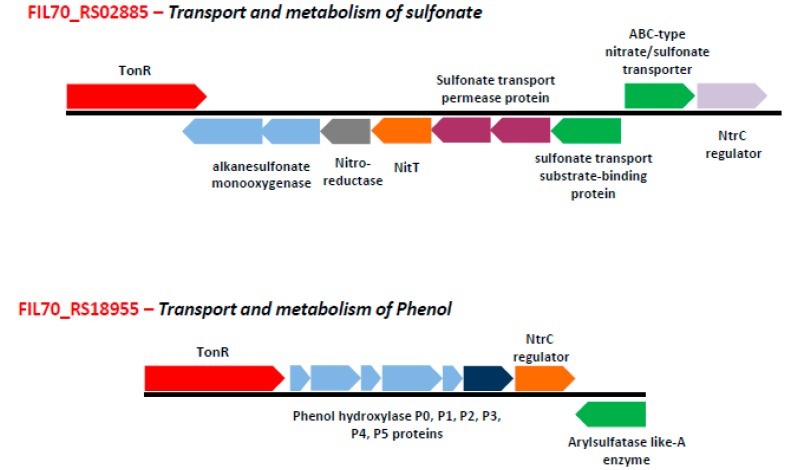
Gene-context analysis of two *_Sf_*TBDT sequences that clustered with xenobiotic responsive TBDTs *Swit_4696* and *Swit_3263* in an expression pattern-based phylogenetic tree.

However, the rest of the 61 putative *_Sf_*TBDTs found in 11 clusters (cluster numbers 1, 2, 3, 9, 10, 11, 12, 13, 14, 15, and 16) of the phylogenetic tree showed no similarity with TBDTs of known function. Transcriptome and proteome analysis was done for certain sphingomonads grown under certain unique physiological conditions. These omics studies showed substrate specific expression patterns of TBDTs [14,16,33,34,36,38,39,40]. About 32 TBDTs showed differential expression in the presence of heavy metals, xenobiotics, temperature stress, and poor nutrient conditions (Table 1). The phylogenetic tree, constructed by including the 61 uncharacterized *_Sf_*TBDTs and the differentially expressed TBDTs of sphingomonads, gave interesting insights into the functions of *_Sf_*TBDTs. The *_Sf_*TBDT (FIL70_RS22795, FIL70_RS21195, and FIL70_RS 11370) clustered with TBDTs of *S. alaskensis* (*Sala_*1228, *Sala_*3108, and *Sala*_0914) involved in the transport of nutrients facilitating its survival under cold stress conditions (Figure 4). Similarly, the *_Sf_*TBDTs (FIL70_RS22300, FIL70_RS01135, FIL70_RS18955, FIL70_RS02885, and FIL70_RS21700) clustered with TBDTs of *S. wittichii* RW1 (*Swit_*4781, *Swit_*4088, *Swit_*3263, *Swit_*4696, and *Swit_*0277) was predicted to be involved in the transport of dibenzo-p-dioxin (DD). These TBDTs of *S. wittichii* were induced in the presence of DD or dibenzofuran (DF). Clustering of *_Sf_*TBDTs with the DD/DF responsive TBDT of *S. wittichii* indicates their role in outer membrane transport of these xenobiotics. Similarly, some *_Sf_*TBDTs (FIL70_RS20305, FIL70_RS06825, FIL70_RS06400, FIL70_RS07020, FIL70_RS06410, and FIL70_RS14490) clustered with sulfanilic acid-responsive TBDTs of *Novosphingobium resinovorans* SA1 (BES08_08830, BES08_18055, BES08_26825, and BES08_18580). Therefore, these *_Sf_*TBDTs are implicated in the transport of sulfonated aromatic amines. Gene context analysis was also carried out for two of the TBDTs (FIL_RS02885 and FIL_RS18955) to examine if these two methods provide identical insights on the functions of TBDTs (Figure 5). These two independent strategies followed indicated substrates only for 29 *_Sf_*TBDTs. Gene context analysis was performed to provide insight on the functional status of the remaining 46 *_Sf_*TBDTs (Table 3). Although experimental evidence is missing to assign a physiological role to a majority of TBDTs, existence of an unusually high number of TBDTs suggests the presence of robust TonB-dependent transport systems in sphingomonads. Such unique transport mechanisms, together with a novel catabolic repertoire, appear to contribute to the survival of sphingomonads in harsh environments.

## Figures and Tables

**Table 1:** Physiological role of TonB-dependent receptors (TBDTs).

Substrates	TBDTs	Genomes	Evidence	References
Iron-siderophore complex	FauA	*Bordetella pertussis*	Ex	[41]
	FecA	*Escherichia coli*	Ex	[42]
	FepA	*Escherichia coli*	Ex	[43]
	FhuA	*Escherichia coli*	Ex	[44]
	FptA	*Pseudomonas aeruginosa*	Ex	[45]
	FpvA	*Pseudomonas aeruginosa*	Ex	[46]
	FrpB	*Neisseria meningitidis*	Ex	[47]
	FyuA	*Yersenia pestis*	Ex	[48]
	HemR	*Yersenia enterocolitica*	Ex	[49]
	TbpA	*Neisseria meningitidis*	Ex	[47]
Cobalamins	BtuB	*Escherichia coli*	Ex	[50]
	BPSL0976	*Burkholderia pseudomallei*	Pr	[51]
	PA1271	*Pseudomonas aeruginosa*	Pr	[51]
	PA2911	*Pseudomonas aeruginosa*	Pr	[51]
	RS02718	*Ralstonia solanacearum*	Pr	[51]
	RSP_2402	*Rhodbacter sphaeroides*	Pr	[51]
Sucrose	SuxA	*Xanthomonas campestris*	Ex	[8]
	Sfri_3988	*Shewanella frigidimarina*	Pr	[8]
Maltose	MalA	*Caulobacter vibrioides*	Ex	[52]
Chitin	XCC2469	*Xanthomonas campestris*	Pr	[53]
	XCC2944	*Xanthomonas campestris*	Pr	[53]
	CPS_1021	*Colwellia psychrerythraea*	Pr	[53]
	Sden_2708	*Shewanella denitrificans*	Pr	[53]
Xylan	XCC4120	*Xanthomonas campestris*	Pr	[8]
Copper	NosA	*Pseudomonas stutzeri*	Pr	[54,55]
	OprC	*Pseudomonas aeruginosa*	Pr	[54,55]
	NosA	*Pseudomonas putida*	Ex	[54,55]
	OprC	*Pseudomonas putida*	Ex	[54,55]
Nickel	FrpB4	*Helicobacter pylori*	Ex	[13]
	Daro_3944	*Dechloromonas aromatica*	Pr	[56]
	BLL6948	*Bradyrhizobium diazoefficiens*	Pr	[56]
	IL54_0463	*Sphingobium* sp. *ba1*	Tr	[16]
	IL54_3057	*Sphingobium* sp. *ba1*	Tr	[16]
Thiamin	BF0615	*Bacteroides fragilis*	Pr	[57]
	CPS_0067	*Colwellia psychrerythraea*	Pr	[57]
	Sputw3181_2365	*Shewanella*	Pr	[57]
	GOX1347	*Gluconobacter oxydans*	Pr	[57]
Cobalt	Daro_1684	*Dechloromonas aromatica*	Pr	[56]
Pectin	XCC0120	*Xanthomonas campestris*	Pr	[8]
Colicin	CirA	*Escherichia coli*	Ex	[58]
Heme	HasR	*Serratia marcescens*	Ex	[49]
	ShuA	*Shigella dysenteriae*	Ex	[59]
Sulfanilic acid	BES08_08830	*Novosphingobium resinovorans SA1*	Tr	[36]
	BES08_18055	*Novosphingobium resinovorans SA1*	Tr	[36]
	BES08_18430	*Novosphingobium resinovorans SA1*	Tr	[36]
	BES08_18580	*Novosphingobium resinovorans SA1*	Tr	[36]
	BES08_22625	*Novosphingobium resinovorans SA1*	Tr	[36]
	BES08_23675	*Novosphingobium resinovorans SA1*	Tr	[36]
	BES08_26825	*Novosphingobium resinovorans SA1*	Tr	[36]
Various substrates under nutrient limitation	*Sala_*0027	*Sphingopyxis alaskensis RB2256*	Tr	[14]
	*Sala_*0914	*Sphingopyxis alaskensis RB2256*	Tr	[14]
	*Sala_*1228	*Sphingopyxis alaskensis RB2256*	Tr	[14]
	*Sala_*1913	*Sphingopyxis alaskensis RB2256*	Tr	[38]
	*Sala_*3029	*Sphingopyxis alaskensis RB2256*	Tr	[14]
	*Sala_*3108	*Sphingopyxis alaskensis RB2256*	Tr	[14]
	Saro_0168	*Novosphingobium aromaticivorans DSM 12444*	Tr	[14]
	Saro_1603	*Novosphingobium aromaticivorans DSM 12444*	Tr	[14]
	SKA58_00625	*Sphingomonas* sp. *SKA58*	Tr	[14]
	SKA58_14617	*Sphingomonas* sp. *SKA58*	Tr	[14]
	SKA58_18137	*Sphingomonas* sp. *SKA58*	Tr	[14]
Dibenzo-p-dioxin	*Swit_*0277	*Sphingomonas wittichii RW1*	Tr	[33]
	*Swit_*1066	*Sphingomonas wittichii RW1*	Tr	[33]
	*Swit_*3263	*Sphingomonas wittichii RW1*	Tr	[33]
	*Swit_*4088	*Sphingomonas wittichii RW1*	Tr	[33]
	*Swit_*4197	*Sphingomonas wittichii RW1*	Tr	[33]
Dibenzofuran	*Swit_*0687	*Sphingomonas wittichii RW1*	Tr	[33]
	*Swit_*2477	*Sphingomonas wittichii RW1*	Tr	[33]
	*Swit_*3189	*Sphingomonas wittichii RW1*	Tr	[33]
	*Swit_*3918	*Sphingomonas wittichii RW1*	Tr	[33]
	*Swit_*4025	*Sphingomonas wittichii RW1*	Tr	[33]
	*Swit_*4696	*Sphingomonas wittichii RW1*	Tr	[33]
	*Swit_*4781	*Sphingomonas wittichii RW1*	Tr	[33]

Ex: experimentally validated, Pr: predicted, Tr: transcriptome and proteome analysis.

**Table 2:** Distribution of TBDTs in sphingomonads.

Name of the Strain	Seq ID	Genome Size (kb)	Phenotype	No. of TBDTs	Refs
*Sphingomonas wittichii RW1*	NC_009511.1	5.38	Dibenzo-p-dioxin	153	[23]
*Sphingobium* sp. *YBL2*	NZ_CP010954.1	4.77	Phenylurea	83	[60]
*Sphingopyxis* sp. *MG*	NZ_CP026381.1	4.15	Organo-phosphate	76	[26]
*Sphingobium fuliginis ATCC 27551*	NZ_CP041016.1, NZ_CP041017.1	5.05	Organo-phosphate	102	[25]
*Sphingobium indicum B90A*	NZ_CP013070.1	3.65	Hexachloro-cyclohexane	45	[61]
*Sphingobium japonicum UT26S*	NC_014006.1, NC_014013.1	4.19	Hexachloro-cyclohexane	66	[62]
*Novosphingobium* sp. *PP1Y*	NC_015580.1	3.9	Fuel oils	48	[63]
*Novosphingobium aromaticivorans DSM 12444*	NC_007794.1	3.5	Phenanthrene	55	[64]
*Sphingobium* sp. *SYK-6*	NC_015976.1	4.2	Lignin	75	[65]
*Sphingobium chlorophenolicum L-1*	NC_015593.1, NC_015594.1	4.45	Pentachlorophenol	99	[66]
*Sphingobium* sp. *22B*	GCA_001580035.1	5.36	Polycyclic aromatic hydrocarbons (PAH)	85	[67]
*Sphingobium* sp. *AM*	GCA_001550165.1	5.31	PAH	86	[68]
*Sphingobium* sp. *ba1*	GCA_000743655.1	4.45	Growth in high nickel ion concentration	75	[16]
*Novosphingobium resinovorans SA1*	NZ_CP017075.1	6.91	Sulfanilic acid	148	[36]
*Sphingopyxis alaskensis RB2256*	NC_008048.1	3.35	Cold marine water	39	[38]
*Sphingomonas wittichii DC-6*	NZ_CP021181.1	5.92	Dibenzo-p-dioxin	141	[69]
*Acinetobacter baumannii AYE*	CU459141.1	3.96	Multi-drug resistance	06	[70]
*Acinetobacter baumannii DS002*	CP027704.1	3.43	Organo-phosphate	05	[32]
*Pseudomonas putida strain JBC17*	CP029693.1	6.84	Dichloro-benzene	14	[71]

**Table 3:** Predicted functions of *_Sf_*TBDTs using gene context analysis.

TBDTs of *S. fuliginis*	Function Based on Gene Context Analysis
FIL70_RS23660	Amino acid transport and metabolism
FIL70_RS20615	Carbohydrate transport and metabolism
FIL70_RS04685	Amino acid transport and metabolism and inorganic ion transport and metabolism (iron)
FIL70_RS06045	Inorganic ion transport and metabolism (iron)
FIL70_RS14890	Nucleotide transport and metabolism
FIL70_RS23225	Lipid transport and metabolism
FIL70_RS08780	Carbohydrate transport and metabolism (xylan)
FIL70_RS20485	Lipid/carbohydrate transport and metabolism
FIL70_RS23345	Carbohydrate/xylulose/xylan transport and metabolism
FIL70_RS21485	Carbohydrate transport and metabolism
FIL70_RS21415	Amino acid/carbohydrate transport and metabolism
FIL70_RS19055	Inorganic ion transport and metabolism (sulfonate)
FIL70_RS18835	Amino acid transport and metabolism/coenzyme transport and metabolism
FIL70_RS11115	Carbohydrate transport and metabolism
FIL70_RS12850	Amino acid transport and metabolism
FIL70_RS19130	Inorganic ion transport and metabolism (iron)/secondary metabolites biosynthesis, transport, and catabolism (sulfonate)
FIL70_RS02520	Nucleotide transport and metabolism
FIL70_RS22785	Inorganic ion transport and metabolism (nickel)
FIL70_RS00175	Inorganic ion transport and metabolism
FIL70_RS22500	Cobalamin transport and metabolism and inorganic ion transport and metabolism
FIL70_RS23300	Lipid transport and metabolism, secondary metabolites biosynthesis, transport and catabolism, inorganic ion transport and metabolism (zinc)
FIL70_RS08535	Lipid transport and metabolism
FIL70_RS12160	Carbohydrate transport and metabolism
FIL70_RS11645	Lipid transport and metabolism
FIL70_RS21180	Lipid transport and metabolism
FIL70_RS21080	Lipid transport and metabolism
FIL70_RS04130	Benzoate transport
FIL70_RS00710	Inorganic ion transport and metabolism and flavin transport and metabolism
FIL70_RS20730	Coenzyme transport and metabolism (flavin transport and metabolism)
FIL70_RS20690	Coenzyme transport and metabolism (flavin transport and metabolism)
FIL70_RS20805	Flavin/secondary metabolite transport and metabolism
FIL70_RS12115	Coenzyme transport and metabolism/secondary metabolite transport and metabolism
FIL70_RS23235	Inorganic ion transport and metabolism
FIL70_RS21680	Lipid transport and metabolism
FIL70_RS03790	Lipid transport and metabolism
FIL70_RS12075	Lipid transport and metabolism
FIL70_RS11540	Lipid transport and metabolism
FIL70_RS08565	Carbohydrate transport and metabolism
FIL70_RS08525	Carbohydrate transport and metabolism
FIL70_RS21930	Lipid transport and metabolism
FIL70_RS22035	Lipid transport and metabolism
FIL70_RS23200	Carbohydrate transport and metabolism
FIL70_RS20020	Inorganic ion transport and metabolism/secondary metabolites biosynthesis, transport, and catabolism
FIL70_RS08430	Coenzyme transport and metabolism/lipid transport and metabolism
FIL70_RS18910	Inorganic ion transport and metabolism
FIL70_RS19370	Lipid transport and metabolism
